# Ginseng glycoprotein and ginsenoside facilitate anti UV damage effects in diabetic rats

**DOI:** 10.3389/fphar.2022.1075594

**Published:** 2022-12-16

**Authors:** Shuang Hu, Lulu Huo, Jing He, Ye Jin, Yongzhi Deng, Da Liu

**Affiliations:** ^1^ School of Pharmacy, Changchun University of Chinese Medicine, Changchun, China; ^2^ Department of Acupuncture and Massage, The Third Affiliated Hospital of Changchun University of Chinese Medicine, Changchun, China

**Keywords:** ginsenoside, ginseng glycoprotein, diabetic, UV damage, peroxide

## Abstract

Diabetes mellitus combined with ultraviolet (UV) radiation damage not only brings great mental stress to patients, but also seriously impairs their quality of life. A UV-irradiated diabetic rat trauma skin model was established by us to investigate the effects and possible mechanisms of ginsenoside and glycoprotein on skin trauma repair in UV-irradiated diabetic rats. In the study, ginsenosides and ginseng glycoproteins were extracted from different parts of ginseng roots. It found that it’s easier to prepare saponins in ginseng bark and proteins in ginseng core in large quantities. Since glycoprotein-like metabolites are relatively novel ginseng extracts, specifically characterized its structures. It was verified that the ginseng glycoproteins are not toxic to HaCaT cells and can significantly increase the survival of HaCaT cells after UV damage at the *in vitro* cellular level. Experiments *in vivo* were conducted to evaluate the therapeutic effects of ginsenoside and ginseng glycoprotein in a rat model of diabetes mellitus combined with UV irradiation injury. Histopathological changes on rat skin after treatment with ginsenoside and ginseng glycoprotein were evaluated by hematoxylin and eosin (H&E) staining and aldehyde fuchsine staining. The expression levels of malondialdehyde (MDA), superoxide dismutase (SOD), glutathione peroxidase (GSH-Px), matrix metalloproteinases (MMPs), hydroxyproline (HYP), interleukin-6 (IL-6), interleukin-1β (IL-1β), and tumor necrosis factor-α (TNF-α) were measured. The results indicate that both ginsenoside and ginseng glycoprotein could improve skin damage and ulcers caused by diabetes combined with UV irradiation and could alleviate a range of skin damage caused by the combination of diabetes and UV irradiation, including peroxidation and collagen fiber loss. Ginsenoside and ginseng glycoproteins can be considered as natural product candidates for the development of new drugs to treat diabetes combined with UV irradiation-induced skin damage.

## 1 Introduction

The development of skin lesions is one of the most common complications of diabetes. When diabetic patients have chronically elevated blood glucose, poor microvascular circulation and poor local cellular function damage all organs of the body, including the skin. Diabetic lesions are widespread and varied, damaging the skin in any part of the body and occurring in all periods of diabetes (Shi et al., 2020). Skin lesions can exacerbate diabetes and lead to severe consequences. In the early stage of diabetes, as blood glucose rises, multiple factors such as the polyol pathway (Garg and Gupta, 2022), diphosphatidylglycerol (DAG)-protein kinase (PK) C pathway (Madhavi et al., 2019), protein non-enzymatic glycosylation (Qais et al., 2021), inflammation (Lotfy et al., 2017), oxidative stress, and insulin resistance interact and accumulate to gradually worsen microvascular endothelial cell damage. Eventually, this leads to the development of diabetic microangiopathy, which causes skin ulceration and damage in diabetic patients (Sandy et al., 2015). People with diabetes can be extremely sensitive to external stimuli due to changes in the skin. Photodamage caused by UV radiation poses a long-term threat and causes many problems for diabetic patients. When UV rays reach the dermis of the skin, they destroy the skin’s collagen and elastin fibers, causing irreversible photodamage. The skin of diabetic patients becomes increasingly fragile after long-term exposure to UV light. In severe cases, epidermal tumors may even develop. Cellular and genetic damage, excessive breakdown of collagen and elastin, peroxidation and the development of acute inflammatory responses have all been associated with skin damage (Yang et al., 2018; Zhou et al., 2019). Therefore, inhibiting these processes may be an effective preventive and therapeutic approach to mitigate skin damage caused by the combination of UV irradiation and diabetes.

Ginseng is a traditional “qi tonic” herb known as the “king of all herbs” (Yin et al., 2021). It has anti-aging, antioxidant, and cell proliferation properties (Attele et al., 1999; Pazyar et al., 2014). As a natural medicine, ginseng has the advantage of low toxicity and low irritation in the treatment of skin injuries (Kim et al., 2019). Ginseng has been reported to promote skin injury healing and improve skin nutrition. Red ginseng oil has been found to protect the skin from UV damage (Truong et al., 2021). The phenolic acid compounds in ginseng root inhibit melanin production and act as skin-brightening agents, treating skin pigmentation disorders caused by abnormal melanin production and melanosome transport (Liu et al., 2021). Extracellular vesicles isolated from ginseng root or ginseng cell culture supernatants have been found to improve the replicative senescence or senescence-associated pigmentation phenotypes of human dermal fibroblasts, or UVB-radiation-treated human melanocytes, by downregulating senescence-associated molecules and/or melanogenesis-associated proteins, respectively (Cho et al., 2021). Currently, it has been suggested that ginsenoside Rc may exert anti-photoaging and barrier function-protective effects in keratinocytes, and thus protect the skin against photooxidative stress induced by exposure to UV radiation (Oh et al., 2017). It has also been suggested that a new role for the ginsenoside RG3 in antiaging *via* mitochondria function in ultraviolet-irradiated human dermal fibroblasts (Lee et al., 2021). And there are few studies on ginseng glycoproteins, which are mainly studied for their role in improving memory function, treating Alzheimer’s disease, protecting the heart, and resisting oligospermia [Luo et al., 2018a; Luo et al., 2018b; Shan et al., 2021 (Chen et al., 2021)]. The mechanism of action of ginsenosides and ginseng glycoprotein on UV-irradiation-induced skin damage in diabetic rats is even less clear. Although a large body of research focuses on skin diseases caused by ultraviolet radiation in sunlight, there is a notable deficiency of credible technical tools and research approaches evaluating the active ingredients of herbal medicine in the field of diabetic dermatopathology. Therefore, elucidating the reparative effect of ginsenoside and ginseng glycoprotein on UV-irradiated diabetic skin wounds is of great significance for the study of diabetic dermatopathology.

## 2 Materials and methods

### 2.1 Reagents and materials

Panax ginseng was purchased from Hunchun Huarui Ginseng Industry Biological Engineering Co., Ltd., Baishan, China. The HaCaT cell was from BeNa Culture Collection (Suzhou, China). Male SD rats (six-week-old, 220 g–230 g, SCXK-2018–0007, Jilin Provincial Laboratory Animal Quality Inspection Center). All experimental procedures performed in this work conformed to the ethical standards of the Medical Ethics Committee of Changchun University of Chinese Medicine, and every effort was made to reduce the number of animals used and any pain and discomfort they experienced. SDS-PAGE gel quick preparation kit (Dalian Meilun Biotech Co., Ltd., Dalian, China), vacuum freeze dryer (Zhengzhou Nanbei Instrument Equipment Co., Ltd., Zhengzhou, China), Sephadex G-75 medium (Jilin JinSai Technology Co. Ltd., Changchun, China), UV-Vis spectrophoto-meter (Shanghai Instrument and Electric Analysis Instrument Co. Ltd., Shanghai, China), HPLC (Shanghai Shimadzu Laboratory Equipment Co.), and Fourier transform infrared spectrometer (BRUKER, Billerica, Massachusetts, United States) were purchased from the respective suppliers. Ginsenoside Rg1 (purity ≥98%) was purchased from Shanghai Yuanye Biotechnology Co. Trypan blue solution (Beijing, China). Chromatography grade methanol and chromatography grade acetonitrile (Fisher, United States). Dulbecco’s modified Eagle’s medium (DMEM) and fetal bovine serum (FBS) were purchased from Gibco (Grand Island, New York, United States). Phosphate buffered solution (PBS), pancreatin, and penicillin–streptomycin solution were purchased from Hyclone (Logan, UT, United States). Cell counting kit-8 (CCK-8) (Wuhan Boshide Biological Engineering Co. Ltd., Wuhan, China) was purchased from the respective suppliers. The ECL chemiluminescence detection kit, SOD, GSH-Px, MDA, and bicinchoninic acid (BCA) were obtained from Beyotime (Shanghai, China). All enzyme-linked immunosorbent assay (ELISA) kits used in this experiment were from Jiangsu MEIMIAN Co., Ltd. (Jiangsu, China). Anti- O-GlcNAc, goat anti-mouse IgG HRP, and goat anti-rabbit IgG HRP antibodies were pur-chased from Cell Signaling Technology (Boston, MA, United States). A microplate reader (Molecular Devices, Silicon Valley, California, United States) and microscope (EVOS XL Core, Thermo Fisher Scientific, Waltham, Massachusetts, United States) were purchased from the respective suppliers.

### 2.2 Determination of saponin content in different parts of ginseng roots

Ginsenosides Rg1, Re, Rf, Rb1, Rc, Rb2, Rb3, Rd was added to methanol and dissolved to make mixed control solutions with concentrations of 101 μg/ml, 103 μg/ml, 102 μg/ml, 103 μg/ml, 101 μg/ml, 98 μg/ml, 98 μg/ml, and 99 μg/ml. The solution was shaken well, then filtered through a 0.22 μm microporous membrane and set aside. Next, 2 g each of ginseng core and ginseng bark powder were weighed to prepare a lyophilized powder of the n-butanol layer in a 2 ml volumetric flask. Methanol was added and dissolved to fix the volume to the scale. The solution was then shaken well and filtered through 0.22 μm microporous membrane. The test solution was obtained from continued filtration.

An Agilent ZORBAX Eclipse XBD-C18 column (4.6 mm × 250 mm, 5 μm) was used; acetonitrile was used as mobile phase A and water as mobile phase B for elution (0 min–30 min, 19% A; 30 min–35 min, 19%–20% A; 35 min–50 min, 20%–26% A; 50 min–55 min, 26%–28% A; 55 min–110 min, 28%–29% A; 110 min–140 min, 29%–33% A). The flow rate was 0.8 ml/min; the detection wavelength was 203 nm; the column temperature was 40°C, and the injection volume was 10 μl.

### 2.3 Extraction of ginseng glycoproteins and study on physicochemical properties and *in vitro* antioxidant activity

#### 2.3.1 Extraction of ginseng glycoproteins

Refer to the previous extraction method and improve it (Fang et al., 2020; Shan et al., 2021). Clean the ginseng root, and separate the ginseng skin and ginseng core by the natural layer ring of the ginseng root. Crush the ginseng skin and ginseng core and set aside. The ratio of ginseng powder to distilled water was 1:15. The extract was shaken twice at 40°C and 120 rpm/min for 12 h. The extract was centrifuged at 4,000 rpm for 30 min, and the supernatant was combined and freeze-dried. The lyophilized ginseng glycoprotein powder was dissolved, added to the dialysis bag, and dialyzed on a magnetic stirrer for 24 h. The external dialysis solution was changed every 6 h. The inner dialysis solution was collected and purified on a Sephadex G-75 column, eluted with distilled water at a flow rate of 1 ml/min; the filtrate was collected, concentrated and dried under reduced pressure to obtain ginseng glycoprotein extract.

#### 2.3.2 Structural identification of ginseng glycoproteins

Glycoproteins are proteins containing sugar chains (Jakab, 2016). O-GlcNAc was detected using western blot analysis to determine the type of protein modifications in the extracts (Saha et al., 2021). Taking 12 μg of ginseng glycoprotein and ginseng skin glycoprotein samples respectively, adding 4 × sample buffer, diluting to 1 ×, mixing with the appropriate protein lysis buffer, and heating at 95°C for 10 min results in a sample concentration of 1 mg/ml–2 mg/ml. The proteins were then separated by SDS-PAGE and transferred to polyvinylidene fluoride (PVDF) membranes by wet transfer under a current of 200 mA. The membrane was then incubated with anti-O-GlcNAc (1:1,000), a primary antibody. After sub-washing, the membranes were incubated with secondary antibodies for 1.5 h at 25°C. Protein blots were detected using the ECL chemiluminescence detection kit, and the exposure time was adjusted to obtain the best signal according to the signal intensity. The results were evaluated using ImageJ software.

The qualitative identification of ginseng glycoproteins was carried out *via* potassium iodide reaction, ninhydrin reaction, Biuret test, Fehling’s reagent reaction, and ferric chloride reaction.

The purity and relative molecular mass of glycoproteins in different parts of ginseng were determined by 12% SDS-PAGE gel electrophoresis and Coomassie Brilliant Blue staining. The parts showing a higher glycoprotein content were used for subsequent experimental studies.

#### 2.3.3 UV spectroscopy

The β-elimination method was used to detect whether there was a change in absorbance value at the UV wavelength of 240 nm. The UV spectra of NaOH-treated ginseng glycoproteins differed from those of non-NaOH-treated glycoproteins. The UV spectra were scanned at 200 nm–350 nm, and the type of glycopeptide bond was determined from the change in absorbance at 240 nm (Yao et al., 2009).

#### 2.3.4 Infrared spectroscopy

Infrared spectroscopy was used to determine the functional group types of the ginseng glycoprotein. A small amount of ginseng glycoprotein and dried potassium bromide were weighed. The sample was ground and mixed with agate mortar, an appropriate amount of the mixture was placed in a compression mold. The sample was pressed into a translucent sheet under a pressure of about 12 MPa. It was then placed in an infrared spectrometer and evaluated by infrared spectroscopy at wavelengths of 4,000 cm^−1^–400 cm^−1^ (blank potassium bromide sheets were pressed down before scanning the sample to remove water and CO_2_ interference).

#### 2.3.5 Amino acid assay of ginseng glycoproteins

The ginseng glycoprotein was placed in a 10 ml hydrolysis tube, to which 6 moL/L hydrochloric acid solution was added. The tube was sealed under a vacuum, placed in a 110°C constant temperature drying oven for 20 h, and then cooled and filtered. After adjusting the pH value and fixing the volume, the assay was conducted.

#### 2.3.6 *In vitro* antioxidant activity of ginseng glycoprotein

Regarding the determination of the ability to scavenge DPPH radicals (Baliyan et al., 2022): The standard ethanol solution of DPPH was prepared, and then ginseng glycoprotein sample solutions with mass concentrations of 200 μg/ml, 400 μg/ml, 600 μg/ml, 800 μg/ml, and 1,000 μg/ml were prepared in ethanol. Together, 100 μl of DPPH free radical solution and 100 μl of the solution were added to a 96-well enzyme labeling plate, mixed well, and kept away from light for 30 min. The absorbance was measured at 517 nm. Vitamin C was used as a positive control in three parallel experiments. The clearance rate is calculated from the following equation:

$$DPPH\;radical\;scavenging\;rate\;\left(\%\right)=1-\frac{\left(A1-A2\right)}{A0}\times 100\%,$$

where A1 is the absorbance measured by mixing different concentrations of sample solution and DPPH solution; A2 is the absorbance measured by mixing different concentrations of sample solution and ethanol solution; and A0 is the absorbance measured by mixing ethanol and DPPH solution.

Regarding the determination of SOD levels: Ginseng glycoprotein sample solutions with concentrations of 200 μg/ml, 400 μg/ml, 600 μg/ml, 800 μg/ml, and 1,000 μg/ml were taken. The kit method was used for the operation, and the SOD enzyme activity of ginseng glycoprotein was determined.

### 2.4 The pharmacological effect of repairing UV damage with ginseng glycoprotein

#### 2.4.1 Cell culture

HaCaT cells were cultured in DMEM supplemented with 10% FBS and 1% penicillin-streptomycin solution at 5% CO_2_ and 37°C. After treatment with DMEM supplemented with 0.5% FBS for 24 h, subsequent experiments were performed.

#### 2.4.2 UV toxicity

UV lamps were obtained from Philips. Cell culture plates were irradiated at a distance of 10 cm from the UV lamp at an irradiation dose of 5 J/cm^2^ (Avadhani et al., 2017). UV intensity was measured by applying a UV meter (Electronic Instrument Factory, Beijing Normal University, China).

UV energy density can be determined from the following equation:

$$E\;\left(J/{\mathrm{cm}}^{2}\right)=t\;\left(s\right)\times P\;\left(W/{\mathrm{CM}}^{2}\right),$$

where E is the UV energy density, t is the time of exposure (in seconds), and *p* is the light intensity.

#### 2.4.3 Cell viability assay

Cell viability was determined by CCK-8 analysis. HaCaT cells were exposed to UV (5 J/cm^2^), and control and blank groups were covered with tin foil. Different concentrations of ginseng glycoprotein (0 μg/ml, 50 μg/ml, 100 μg/ml, and 200 μg/ml) were given for 24 h after modeling. After culturing HaCaT cells in 96-well plates (5 × 10^3^ cells per well), 10 μl of CCK-8 solution was added to each well at a 10% dilution. The cells were then incubated for 1 h, and the mean optical density (OD) was measured at 450 nm using a microplate reader. The cell viability percentage was calculated according to the following formula:

$$Percentageofcellviability=\frac{\text{OD}\text{treatment}\text{group}}{\text{OD}\text{control}\text{group}}\times 100\%.$$

all experiments were performed in triplicate.

### 2.5 Wound healing and cell proliferation assay

HaCaT cells were inoculated in six-well plates at a density of 5 × 10^5^ cells and cultured overnight. The HaCaT cells were subsequently exposed to UV (5 J/cm^2^). A 10 μl pipette tip was used to produce uniform scratches. Next, 50 μg/ml ginseng saponin and ginseng glycoprotein were added to serum-free DMEM and incubated with the cells for another 24 h. Migration curves were recorded using an inverted fluorescence microscope, and the scratched area was measured using ImageJ software.

$$\text{Healing}\;\text{rate}=\frac{\left(0h\;\text{wound}\;\text{area}-24h\;\text{wound}\;\text{area}\right)}{0h\;\text{wound}\;\text{area}}\times 100\%$$

All experiments were performed in triplicate.

### 2.6 Effects of ginsenoside and ginseng glycoprotein on skin damage in rats with diabetes combined with UV irradiation

Male SD rats were kept under a light/dark cycle at 20°C–25°C for 12 h with free access to food and water. Rats were allowed to acclimatize to the environment for one week before the start of the experiment. The rat diabetes model was first established by intraperitoneal injection of streptozotocin (STZ) 55 mg/kg diluted by citrate buffer, and blood glucose was tested daily after the injection. When the blood glucose value was greater than 13.3 mol/L, and histopathological examination of the pancreas confirmed that the islet cells had been destroyed, the model was successfully established.

One month after the successful establishment of the diabetes model, the diabetes model group was randomly divided into five subgroups of eight animals each: a blank control group (diabetes, no UV irradiation, no treatment), a model group (diabetes, UV irradiation, no treatment), a VE group (diabetes, UV irradiation, treatment), a ginsenoside administration group (diabetes, UV irradiation, treatment), and a ginseng glycoprotein administration group (diabetes, UV irradiation, treatment). The hair on the back of the rats was removed from an area of about 3 cm × 3 cm. The rats were anesthetized by intraperitoneal injection of 3% sodium pentobarbital (50 mg/kg). Ultraviolet light (UVA + UVB) was applied to irradiate the naked back of the rats to establish a skin photodamage model of diabetic rats (Jin et al., 2022).

Except for the control group, the rats in each group had their back hair shaved and then irradiated under a UV lamp at a fixed distance of 20 cm for three weeks. In the first week, the rats were irradiated for 20 min per day; in the second week, the dose was increased to 40 min per day; and in the third week, the irradiation time was increased to 60 min per day with approximately 40% increase in UV dose to avoid acute damage. During this period, there was no change in any irradiation conditions, except for the change in irradiation duration. The control group did not receive irradiation. UV irradiation was stopped when the skin on the back of the rats showed redness, crusting, and local ulceration, indicating that a diabetic rat skin photodamage model had successfully been established. The establishment time of the UV damage model can be adjusted according to the rate of skin damage, which may differ among individual diabetic rats. Treatment administration was performed after cessation of UV irradiation. The dose administered to the treatment group was calculated based on the equivalent human-to-rat dose ratio of bovine serum albumin. The calculation formula was as follows:

$$\text{DB}\;\left(\frac{g}{\text{kg}}\right)=\text{DA}\times \frac{\text{RB}}{\text{RA}}\times {\left(\frac{\text{WA}}{\text{WB}}\right)}^{\frac{1}{3}},$$

where DB is the rat dose, DA is the human dose, and WA and WB are the human and rat body weights, respectively. RA and RB are the constants (body factor) for humans and rats, respectively:

$$\begin{array}{c} \text{DB}\left(\frac{g}{\text{kg}}\right)=\text{DA}\times \frac{0.06}{0.1}\times {\left(\frac{70}{0.2}\right)}^{\frac{1}{3}},\\ \text{DB}\left(\frac{g}{\text{kg}}\right)=\text{DA}\times 6.3. \end{array}$$

The equivalent dose ratio between human and rat is about 6.3, and the equivalent human-rat dose ratio used in this study is 7. According to the 2020 edition of the Chinese Pharmacopoeia, the recommended daily dose of ginseng for humans is 9 g/day, so the daily dose for rats was calculated at 1.05 g/kg. In order to select the appropriate dose to be administered, two doses (1 × and 3 × of the reference calculation) were used in the preliminary experiments. Based on the preliminary experiments, the three-fold reference dose was ultimately selected. Thus, the equivalent triple dose for diabetic rats in the ginsenoside and ginseng glycoprotein groups was calculated to be 3.15 g/(kg day). The VE group was given the same dose, and the blank group was coated with saline. The skin condition of rats in each group was observed after five days of continuous administration (Filip et al., 2013). Five groups of rats were executed after anesthesia, and the exposed target skin was removed and fixed with 4% paraformaldehyde and paraffin embedding to study changes to the skin tissue. Cytokine levels in skin tissues were measured. Finally, skin tissues were stained with H & E and aldehyde fuchsine staining to analyze the histology of the epidermis, dermis, collagen fibers, and elastic fibers.

### 2.7 Statistical analysis

All experiments were conducted at least three times. Data are shown as the mean ± standard deviation. All values were analyzed using one-way ANOVA (Student-Newman–Keuls tests). Tukey’s test was used for pair comparisons between groups. The GraphPad Prism9 software was used for statistical analysis and mapping. Statistical significance was set at *p* < 0.05.

## 3 Results

### 3.1 Determination of saponin and glycoprotein content in different parts of ginseng roots

**FIGURE 1:**
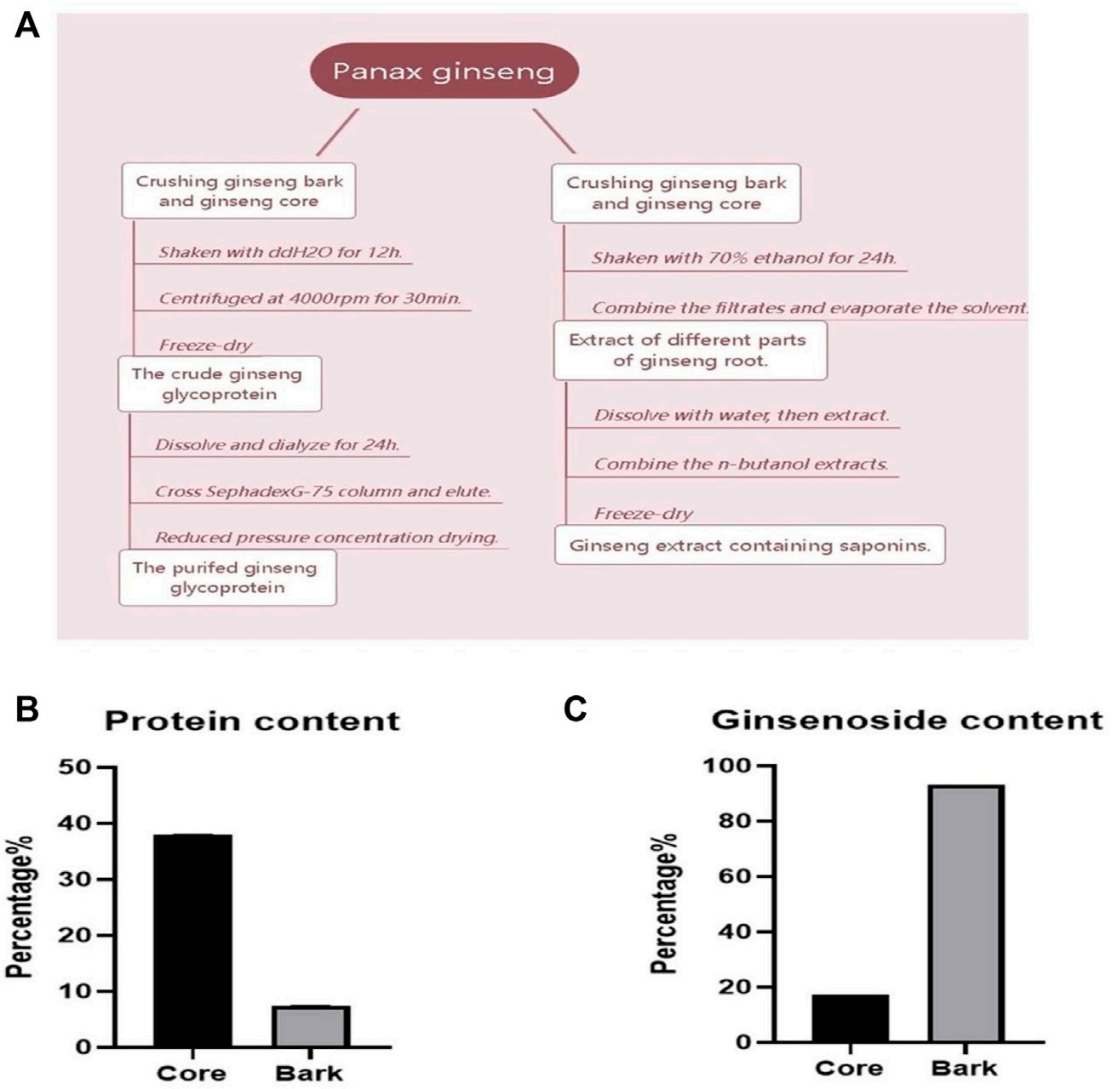
Determination of saponin and glycoprotein content in different parts of ginseng roots. **(A)** Flow chart of ginsenoside and ginseng glycoprotein extraction from different parts; **(B)** Percentage of saponins content in different parts of ginseng roots; **(C)** Percentage of glycoprotein content in different parts of ginseng roots.

The process of extracting saponins and glycoproteins from different parts of ginseng roots is shown in Figure 1A. The contents of ginsenosides Rg1, Re, Rf, Rb1, Rc, Rb2, Rb3, and Rd in ginseng core and ginseng bark were measured as shown in Table 1. The total amount of the eight saponins in the ginseng bark was found to be 5.41 times higher than that in the ginseng core. The percentage of ginsenosides in the ginseng bark extract reached 93.22%, whereas the percentage of saponins in the ginseng core extract reached 17.23% (Figure 1B). Using protein content as the test standard, the protein content in ginseng core reached 38.09%, while the protein content in ginseng skin was only 7.46% (Figure 1C). Therefore, ginsenosides extracted from ginseng bark and glycoproteins extracted from ginseng core were selected for the follow-up experiments.

**TABLE 1:** Content of 8 saponin in ginseng core and ginseng bark (%) (x ± s, *n* = 6).

Saponin	Ginseng core (%)	Ginseng bark (%)
Rg1	0.1459 ± 0.0332	0.2635 ± 0.0233
Re	0.0227 ± 0.0121	0.4091 ± 0.0102
Rf	0.0190 ± 0.0012	0.0825 ± 0.0046
Rb1	0.1008 ± 0.0053	0.5024 ± 0.0023
Rc	0.0172 ± 0.0051	0.2604 ± 0.0037
Rb2	0.0132 ± 0.0301	0.2041 ± 0.0217
Rb3	0.0029 ± 0.0517	0.0632 ± 0.0384
Rd	0.0229 ± 0.0036	0.0792 ± 0.0218

### 3.2 Structure and physicochemical properties of ginseng glycoproteins

**FIGURE 2:**
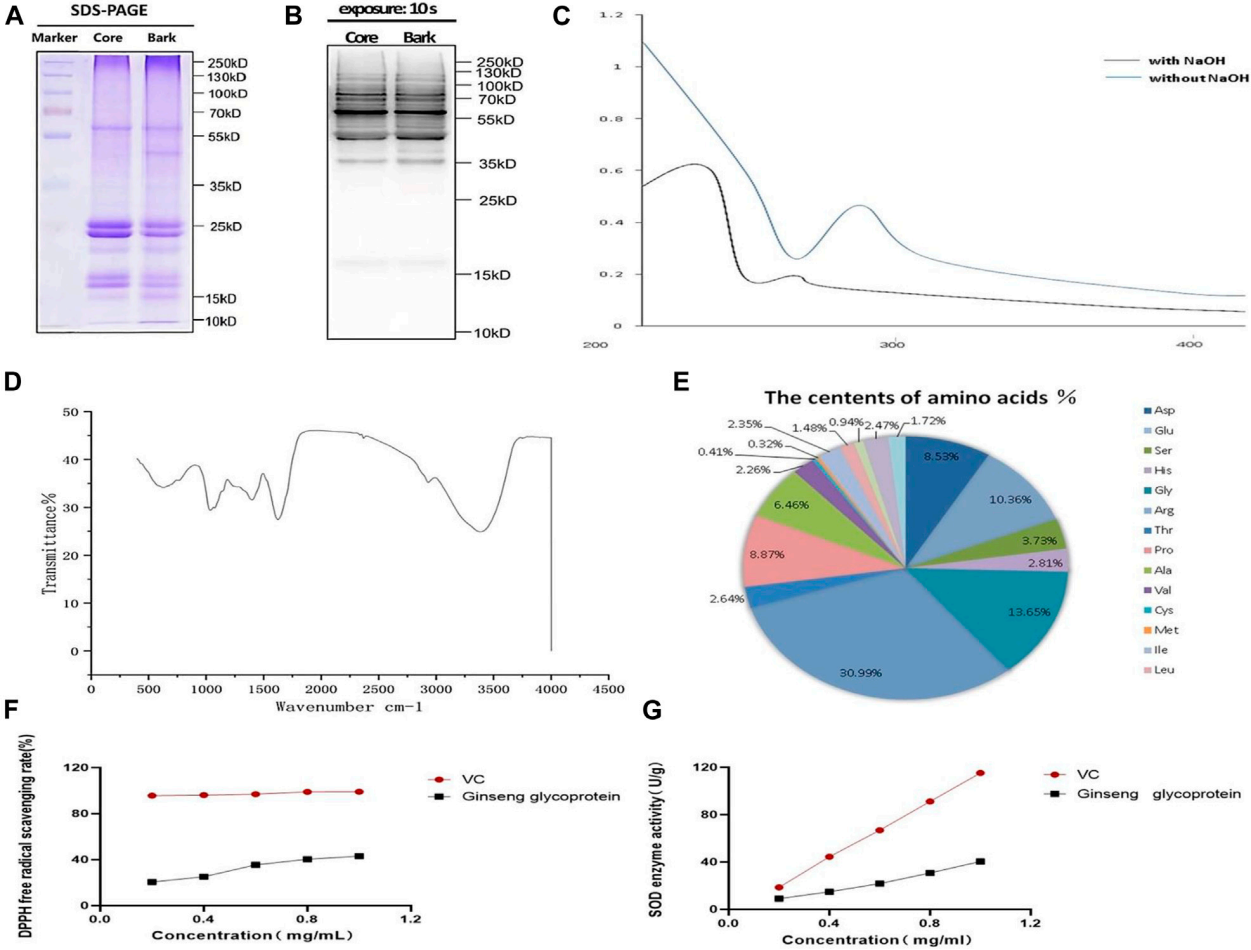
Structure and physicochemical properties of ginseng glycoproteins. **(A)** The results of Komas Brilliant Blue staining of ginseng extract samples; **(B)**; Western blotting assay of anti-O-GlcNAc **(C)** UV spectra of ginseng glycoproteins; **(D)** Infrared spectra of ginseng glycoprotein; **(E)** Amino acid composition of ginseng glycoprotein; **(F)** Comparison of the DPPH radical scavenging rate of ginseng glycoprotein and VC; **(G)** Comparison of SOD expression of ginseng glycoprotein and VC.

After applying Coomassie Brilliant Blue staining, the molecular weights of ginseng glycoproteins were found to be mainly concentrated between 17 and 35 KD by (Figure 2A). The results of western blotting with pan anti-O-GlcNAc antibody are shown in Figure 2B. It was found that the protein of ginseng glycoprotein was modified by oxygen glycosylation to form glycoprotein. The reaction of iodine potassium iodide showed no color change, indicating that the ginseng glycoprotein was a non-starch polysaccharide; the reaction of Biuret test turned blue-purple, indicating the presence of amino acids or peptides; the reaction of double-bonded urea reagent was blue, indicating the presence of proteins; and the reaction of iron reagent with ferric chloride was negative, indicating the absence of monosaccharides and polyphenols.

The UV spectrum of ginseng glycoprotein was analyzed by dissolution in 10 ml of 0.4 mol/L NaOH (Figure 2C). The ginseng glycoprotein solution without NaOH treatment had a clear absorption peak at 280 nm, indicating the presence of protein. The addition of dilute NaOH facilitated the conversion of serine linked to the sugar chain into α-aminoacrylic acid and threonine into α-aminobutyric acid, constituting unsaturated amino acids with significant absorption at 240 nm. Thus, an O-glycopeptide bond type was determined.

According to the infrared spectra that had been obtained, the samples were found to contain polysaccharide and protein molecules (Figure 2D). The samples were also found to contain stretching vibrational features of methyl, methylene, and other alkyl groups. In addition, the amide group showed an absorption peak at 1,640 cm^−1^, and the sulfate group showed a stretching vibration feature at 1,400 cm^−1^. It can be concluded that the ginseng glycoprotein sample contains polysaccharides, proteins, methyl, methylene, and other alkyl groups, in addition to the amide group and pyranose structure.

Additionally, as shown in Figure 2E, the ginseng glycoprotein was found to consist of 17 amino acids: Asp (8.43%), Glu (10.36%), Ser (2.73%), His (2.81%), Gly (12.65%), Arg (29.95%), Thr (2.27%), Pro (8.87%), Ala (5. 46%) Val (2.06%), Cys (0.41%), Met (0.22%), Ile (2.25%), Leu (1.33%), Phe (0.84%), Lys (2.47%), and Tyr (1.72%).

DPPH is a relatively stable free radical that is widely used to determine the antioxidant capacity of polysaccharide samples (Zeng et al., 2013). Different concentrations of ginseng glycoprotein all showed scavenging effects on DPPH radicals, and a significant quantitative-effect relationship was observed in the experimental concentration range (Figure 2F). The greatest scavenging of DPPH radicals was achieved when the ginseng glycoprotein concentration reached 1 mg/ml, or 43.06%, which was lower than that of the positive control drug VE. Figure 2G, shows that the higher the ginseng glycoprotein content, the higher the SOD expression. When the ginseng glycoprotein concentration reached 1 mg/ml, the SOD enzyme activity reached 40.44 (U/g).

### 3.3 Medicinal effect of ginseng glycoprotein in repairing UV irradiation

**FIGURE 3:**
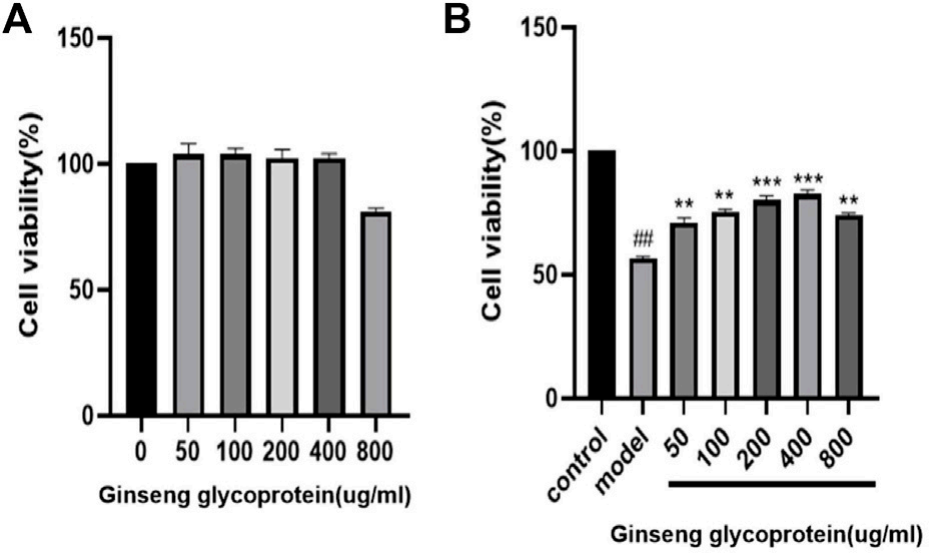
Ginseng glycoprotein has a pharmacological effect on the repair of UV irradiation. **(A)** Effect of ginseng glycoprotein on HaCaT cell viability; **(B)** Effect of ginseng glycoprotein on UV-induced HaCaT cell viability.

To investigate whether ginseng glycoprotein has toxic effects on HaCaT cells, we used the CCK-8 method was used to detect the effect of ginseng glycoprotein on the viability of HaCaT cells. As shown in Figure 3A, no significant difference was found in the viability of HaCaT cells treated with different concentrations of ginseng glycoprotein compared with the control group. The results indicate that ginseng glycoprotein has no toxic effect on HaCaT cells.

Meanwhile, the effect of ginseng glycoprotein on the viability of UV-induced HaCaT cells before and after treatment was examined using the CCK-8 method. As shown in Figure 3B, the cell survival rate was significantly lower in the model group than in the control group (*p* < 0.01), which suggests successful modeling. The addition of different concentrations of ginseng glycoprotein in the drug intervention group could alleviate the reduction of cell viability caused by UV irradiation in a concentration-dependent manner (*p* < 0.05, *p* < 0.01). This demonstrates the significant efficacy of ginseng glycoprotein at the cellular level *in vitro*.

### 3.4 Promotion of HaCaT cell migration with ginsenosides and ginseng glycoprotein

**FIGURE 4:**
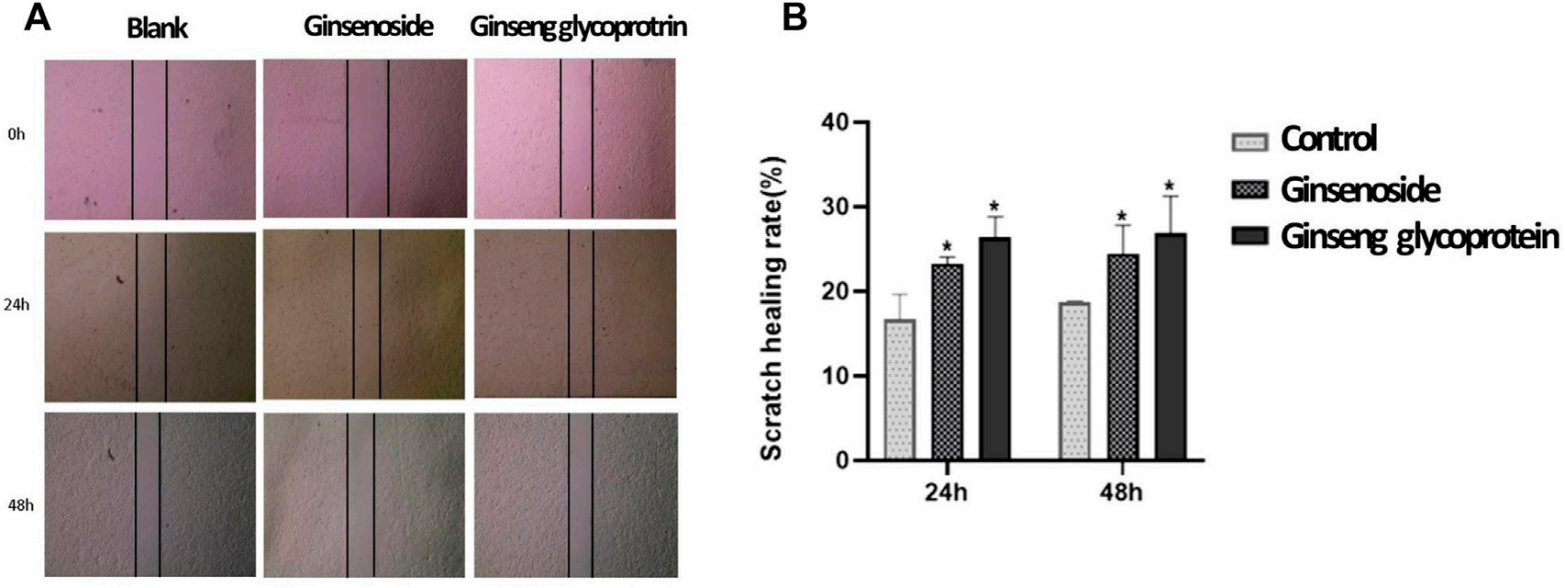
Ginsenoside and ginseng glycoprotein can promote HaCaT cell migration. **(A)** Ginsenoside and ginseng glycoprotein promote HaCaT cell migration. Scale bar: 200 μm; **(B)** Quantitative analysis *p < 0.05, indicating a significant difference between control and treatment groups. Data are expressed as *N* = 3.

A wound-healing assay was applied to assess the effect of ginsenosides and ginseng glycoprotein on the migration properties of HaCaT cells *in vitro*. The changes in the scratch area of each group were observed at 24 h and 48 h to assess HaCaT cell migration (Figures 4A,B). The results show that ginseng glycoprotein promotes the migration of HaCaT cells more prominently than ginsenoside.

### 3.5 Using ginsenosides and ginseng glycoprotein to improve skin damage caused by diabetes combined with UV irradiation in rats

#### 3.5.1 Skin wound repair in diabetic rats with ginsenosides and ginseng glycoprotein

**FIGURE 5:**
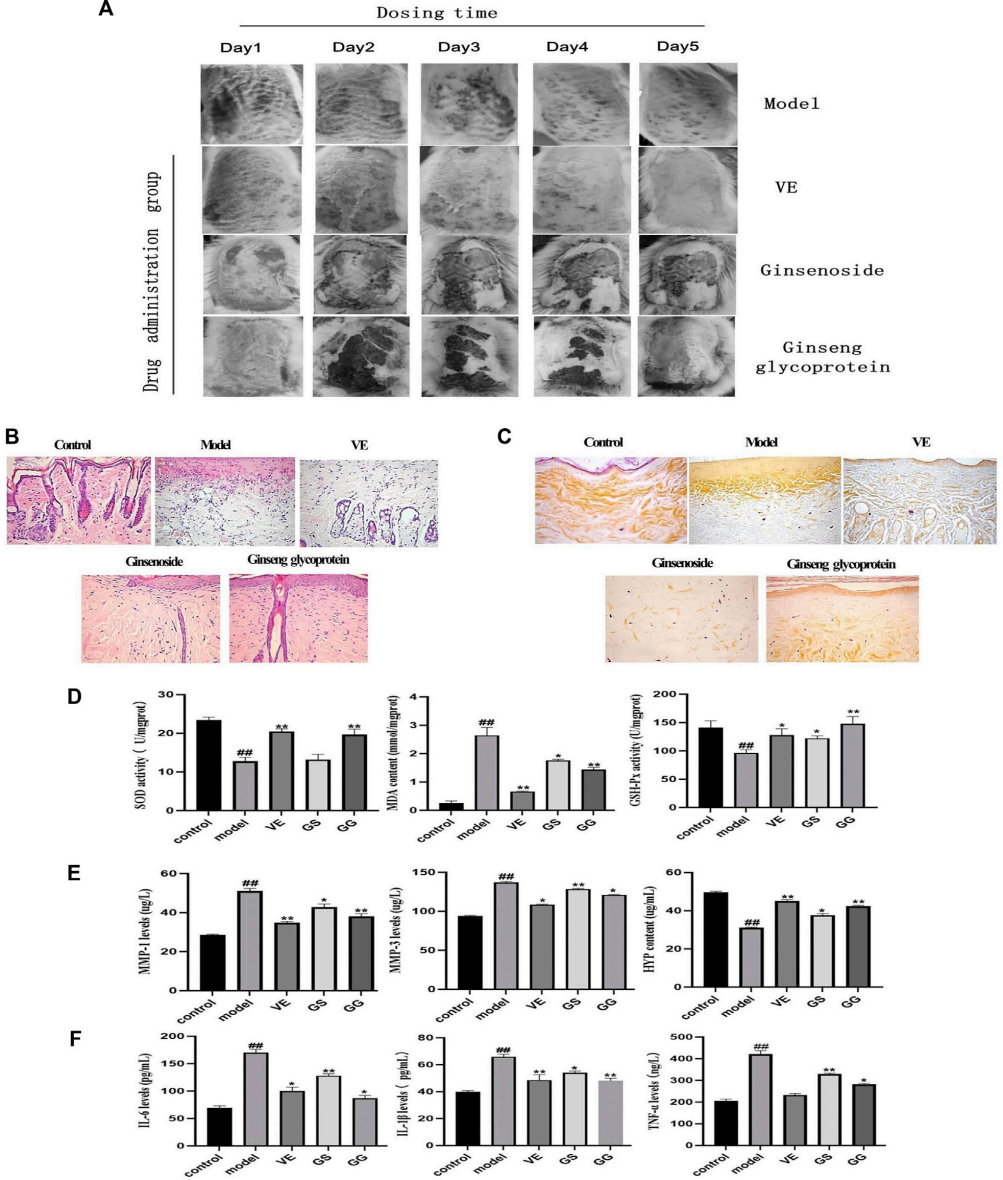
Ginsenoside and ginseng glycoprotein can improve skin damage caused by diabetes combined with UV irradiation in rats. **(A)** Changes in the appearance of diabetic rats after 5 days of continuous dosing; **(B)** Histology of skin by H & E staining of the skin; tissue section (× 200); **(C)** Histology of skin by aldehyde fuchsine stains of the skin tissue section (× 200); **(D)** Expression of MDA, SOD and GSH-Px in the skin homogenates of diabetic rats in different groups; **(E)** The expression of matrix metalloproteinase 1 (MMP-1), matrix metalloproteinase 3 (MMP-3) and hydroxyproline (HYP) in rat skin tissues was detected by enzyme-linked immunosorbent assay (ELISA); **(F)** The expression of tumor necrosis factor-α (TNF-α), interleukin-1β (IL-1β) and interleukin-6 (IL-6) in rat serum was measured by enzyme-linked immunosorbent assay (ELISA). Ginsenoside (GS), Ginseng glycoprotein (GG) #*p* < 0.05 ##*p* < 0.01 ###*p* < 0.001 *versus* control group; *p < 0.05 **p < 0.01 ***p < 0.001 *versus* model group. Data are expressed as mean values ± SD (*n* = 8).

The appearance of the diabetic rats after UV irradiation is shown in Figure 5A. Compared with the control group, the skin on the back of the diabetic rats showed obvious redness, crusting, and ulceration after UV irradiation. Compared with the model group, skin damage improved in all groups after treatment administration. In addition, hematoxylin and eosin (H&E) staining results showed (Figure 5B) that skin cells in the control group were neatly arranged without hemorrhage, edema, or other abnormalities. UV exposure leads to skin cell damage with a large infiltration of inflammatory cells, thickening of the stratum corneum, and deterioration of skin condition. In the model group, the stratum corneum was significantly thickened, the dermis was infiltrated with inflammatory cells, collagen fibers were absent, and the junction between epidermis and dermis was flattened. These symptoms of UV irradiation were significantly improved by the addition of ginsenoside, ginseng glycoprotein, and VE. The epidermal thickness of the skin of rats in the ginseng glycoprotein group was thin and close to normal, with a small amount of inflammatory cell infiltration at the junction of the epidermis and dermis; the collagen fibers in the dermis near the epidermis were slender and regularly arranged, with the direction parallel to the epidermis. The skin thickness of the ginsenoside group was relatively thin; similarly, there was a small amount of inflammatory cell infiltration at the epidermal-dermal junction. The improvement effect was slightly worse than that of the glycoprotein group. The improvement in the VE group was consistent with that of the ginseng glycoprotein group. No effect was observed in animals treated with the 0.9% saline control solution.

Aldehyde fuchsine staining was used to observe changes in skin tissue’s elastic fibers and assess skin elasticity (Figure 5C). In the control group, the elastic fibers showed a relatively clear reticular structure, and the collagen fibers were coarse. In the model group, the dermal collagen and elastic fibers were significantly reduced and irregularly arranged, with obvious clusters and accumulations. All treatment groups showed different degrees of improvement. Compared with the model group, the rats in the ginseng glycoprotein group had a clear dermal collagen fiber network structure with a thicker shape. The structure of the dermal collagen fiber network in the ginsenoside group was not sufficiently well-defined, and the improvement of elastic fiber changes in the VE group was consistent with that of the ginseng glycoprotein group.

#### 3.5.2 Effects of ginsenosides and ginseng glycoprotein on MDA, SOD, and GSH-Px expression

Superoxide dismutase (SOD), glutathione peroxidase (GSH-Px), and malondialdehyde (MDA) play important roles in the overall antioxidant defense network. MDA is the main product of lipid peroxidation and can be used to assess oxidative skin damage. SOD and GSH-Px can also be used to assess oxidative skin damage after UV exposure. MDA, SOD, and GSH-Px were measured in the skin tissue homogenates of rats in each group. Compared with the control group, the expression of MDA in the skin tissue of the model group was significantly increased, while the expression of SOD and GSH-Px decreased. In contrast, ginseng glycoprotein significantly reduced MDA levels and significantly increased SOD and GSH levels in rat skin tissues. Ginsenosides did not affect these three cytokines as much as the ginsenoside glycoproteins. Therefore, ginsenoside glycoprotein can ameliorate the oxidative damage induced by UV radiation (Figure 5D).

#### 3.5.3 Effect of ginsenosides and ginseng glycoprotein on MMPs and HYP expression

Research shows that MMP-1 can degrade type I and type III collagen in the extracellular matrix, and MMP-3 can digest type IV collagen in the basement membrane and activate MMP-1 precursors to form MMP-1. MMP-3 synergizes with MMP-1 to degrade collagen and elastin, leading to collagen and elastin rupture (Lee et al., 2021; Wan et al., 2021). Therefore, this study assessed the expression of MMP-1 and MMP-3. As can be seen, MMP-1 and MMP-3 expression significantly increased in the model group compared to the control group. In contrast, the expression of MMP-1 and MMP-3 significantly decreased in each treatment group.

Hydroxyproline (HYP), a unique amino acid in collagen, is another primary metabolite of collagen tissue. The detection of HYP can show the collagen catabolism/metabolism of skin tissue. Compared with the control group, the content of HYP in the skin tissue of rats decreased after UV irradiation and increased after treatment with ginsenosides and ginseng glycoprotein. However, it is clear from the data that ginsenosides are much less effective than ginsenoside glycoproteins. Ginseng glycoprotein has been shown to block collagen decomposition effectively (Figure 5E).

#### 3.5.4 Effect of ginsenosides and ginseng glycoprotein on IL-6, IL-1β, and TNF-α expression

The increased expression of pro-inflammatory cytokines and decreased expression of anti-inflammatory cytokines were found in the ulcerated tissues of UV-irradiated diabetic rats (Zhou et al., 2021). The levels of TNF-α, IL-6, and IL-1β were significantly higher in the model group compared with the control group. TNF-α, IL-6, and IL-1β levels decreased in the ginsenosides and ginseng glycoprotein groups. Notably, ginseng glycoproteins were more effective than ginsenosides in reducing excessive inflammatory responses in a diabetic rat model of UV damage. Ginseng glycoprotein could reduce the inflammatory response in the skin tissue of diabetic rats after UV irradiation by inhibiting the release of inflammatory factors (Figure 5F).

## 4 Discussion

Ginsenosides and ginsenoside glycoproteins were extracted *via* the shaking method, which could circumvent the adverse effects of the active ingredients of ginseng compared with the conventional hot water extraction method and the water bath reflux extraction method. Shaking is beneficial for the dissolution of the active ingredients, which makes full use of ginseng as a traditional Chinese medicine resource in a simple experimental operation. As the results showed, ginseng skin contains more saponins and the ginseng core contains more glycoproteins. This finding facilitates the preparation of ginsenosides and ginseng glycoproteins in large quantities. In addition, a secondary extraction was performed on remaining ginseng root residue after ginsenoside extraction, and it turned out that the extract of ginseng root residue still contained glycoproteins. This discovery helps to realize the circular extraction of ginseng and make full use of its medicinal resources, while facilitating industrial production. It is worth mentioning that only the skin and core of fresh ginseng roots can be separated more easily due to the presence of the laminar ring structure; therefore, the extraction of ginseng skin and ginseng core is limited by the season and needs more sophisticated extraction strategies to overcome seasonal constraints. The ginsenosides and ginseng core glycoproteins we extracted are both mixtures, and their compositions and ratios are not stable, especially the active ingredients that need to be further clarified. We will continue to explore the extraction strategy of ginseng and try to screen for biologically active saponin monomers and small molecule peptide monomers, so as to further investigate their biological activities in depth.

Since there are relatively more studies on the pharmacological efficacy of ginsenoside, and fewer on ginseng glycoproteins as novel ginseng extracts, it was necessary to undertake the structural identification of the extracted ginseng glycoprotein-like substances. The extracts were analyzed to detect the types of protein modifications present, and the proteins in the ginseng glycoproteins were found to be modified by oxygen glycosylation to form glycoproteins. UV detection indicated an O-glycosidic bond type and the presence of pyranose conformation in the sugar chain. Extracted ginseng glycoprotein, with a molecular weight of around 17–35 KD, is considered a large molecule of Chinese medicine. Due to ginseng glycoprotein’s complex structure and susceptibility to temperature, acidity, and alkalinity, its structure is easily altered. This reduces its biological activity, which makes it difficult to conduct basic research and clinical applications. Meanwhile, to address the half-life and targeting of natural macromolecule products *in vivo*, further drug delivery and release studies can be conducted. It is of great significance for the development and application of traditional Chinese medicine macromolecules to make full use of new technology to develop new dosage forms, improve drug delivery systems, assess their advantages, and explore new drug delivery routes. Future research should continue to investigate, for example, the stability and drug effects of novel formulations of liposomes encapsulating natural macromolecules such as glycoproteins and polysaccharides.

UV irradiation has a severely damaging effect on the skin of diabetic patients. It found that high levels of inflammatory response and oxidative stress are closely related to diabetes and UV irradiation-induced skin damage, while ginsenosides and ginseng glycoproteins have been reported to play an important role in regulating inflammation and immune modulation (Lorz et al., 2020; Chen et al., 2021). For epidermal keratin-forming cells, various extracellular stimuli (including UV light) can trigger signaling cascades associated with stress responses, the most important of which is the activation of mitogen-activated protein kinases (MAPKs). In previous reports, UVB irradiation can stimulate MAPKs by activating the phosphorylation of c-Jun N-terminal kinase (JNK), p38, and extracellular signal-regulated kinase (ERK) (Chen et al., 2019; Tang et al., 2019; Jo et al., 2020). Therefore, antioxidants may be an effective way to intervene in complications and comorbidities of diabetes, which is the potential for future studies to investigate the effect of the MAPK pathway on UV damage in diabetic rats.

**FIGURE 6:**
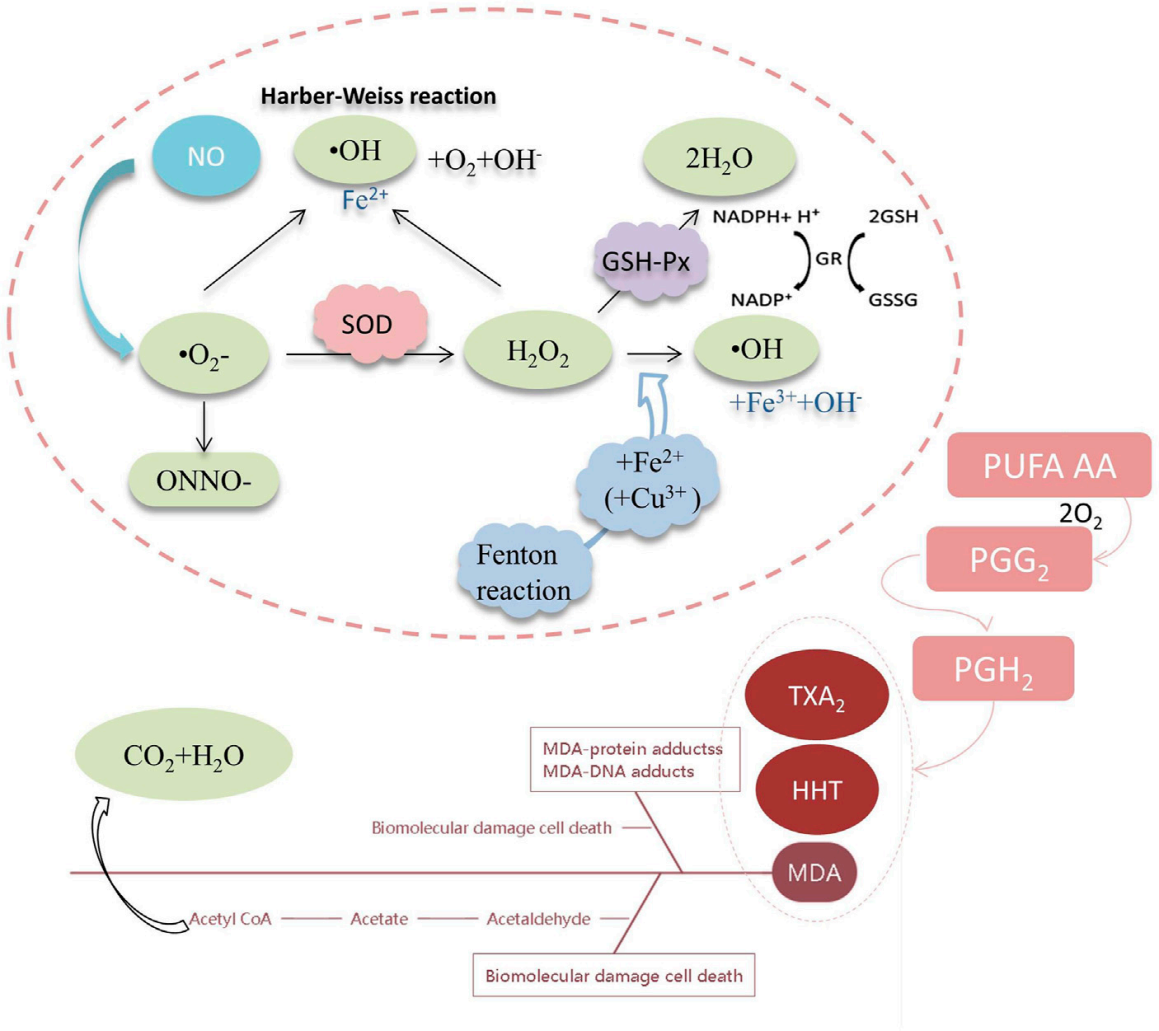
MDA, SOD and GSH-Px are involved in the oxidation process.

Cytokines are central to the wound-healing process. Important antioxidant enzymes include superoxide dismutase (SOD) and glutathione peroxidase (GSH-Px). SOD is responsible for the disproportionation of superoxide anions to hydrogen peroxide, while GSH-Px enzymes can reduce hydrogen peroxide and thereby prevent the production of highly toxic hydroxyl radicals (Figure 6). Unsaturated fatty acids form conjugated dienes at the initial stage of lipid oxidation triggered by free radicals and some oxidation. They then form cyclic peroxides, and the final major product is malondialdehyde (MDA). Meanwhile, GPx enzymes can reduce the hydroperoxides of polyunsaturated fatty acids. To determine the extent of lipid oxidative damage in the body or cells, the activity of antioxidant enzymes (SOD, GPx) and the level of lipid peroxidation product (MDA) are measured. This determines the severity of cellular attack by free radicals (Çavuşoğlu et al., 2022). In a diabetic rat skin UV damage model, this study found that ginseng glycoproteins have superior reparative efficacy compared to ginsenosides. Ginseng glycoprotein could effectively inhibit lipid peroxidation and increase the expression of SOD, GSH-Px, and MDA in skin cells. Moreover, ginseng glycoprotein was found to reduce the expression of MMPs, HYP, and inflammatory factors in skin tissues, achieving anti-inflammation and enhancing skin elasticity while exerting the therapeutic effect of repairing UV wounds in diabetic rats. Our experimental results demonstrate that ginsenosides and ginseng glycoproteins have some therapeutic effects on UV-induced skin damage in rats, but their complex action mechanisms of action need to be elaborated by extensive experimental data. In the future, it is recommended to continue to study the effects of ginsenosides and glycoproteins on nucleic acid metabolism and signaling pathways, including the study of kinases, receptors, protein metabolism.

Research and development of innovative drugs based on ginsenoside ginseng have been reported (Yu et al., 2019; Jung et al., 2021; Yang et al., 2022). For ginseng glycoproteins, despite their use in improving memory function, treating Alzheimer’s disease, protecting the heart, and fighting oligospermia, they are cross-species exogenous proteins for humans, and it needs further study their effects on the immune system, especially allergic and rejection reactions, need further study. In conclusion, this study establishes the foundation for the development of new drugs from natural macromolecular products, as well as provides the basis to analyze the effects of ginseng glycoproteins on the treatment of diabetes combined with UV irradiation trauma. Ginseng shows great potential for antioxidation and improvement of skin conditions.

## Data Availability

The original contributions presented in the study are included in the article/Supplementary Material, further inquiries can be directed to the corresponding authors.
