# Supplementary figures and images for: Ginseng glycoprotein and ginsenoside facilitate anti UV damage effects in diabetic rats
Source: Front Pharmacol. 2022 Dec 16;13:1075594. doi: 10.3389/fphar.2022.1075594 (PMC9800513; doi:10.3389/fphar.2022.1075594)

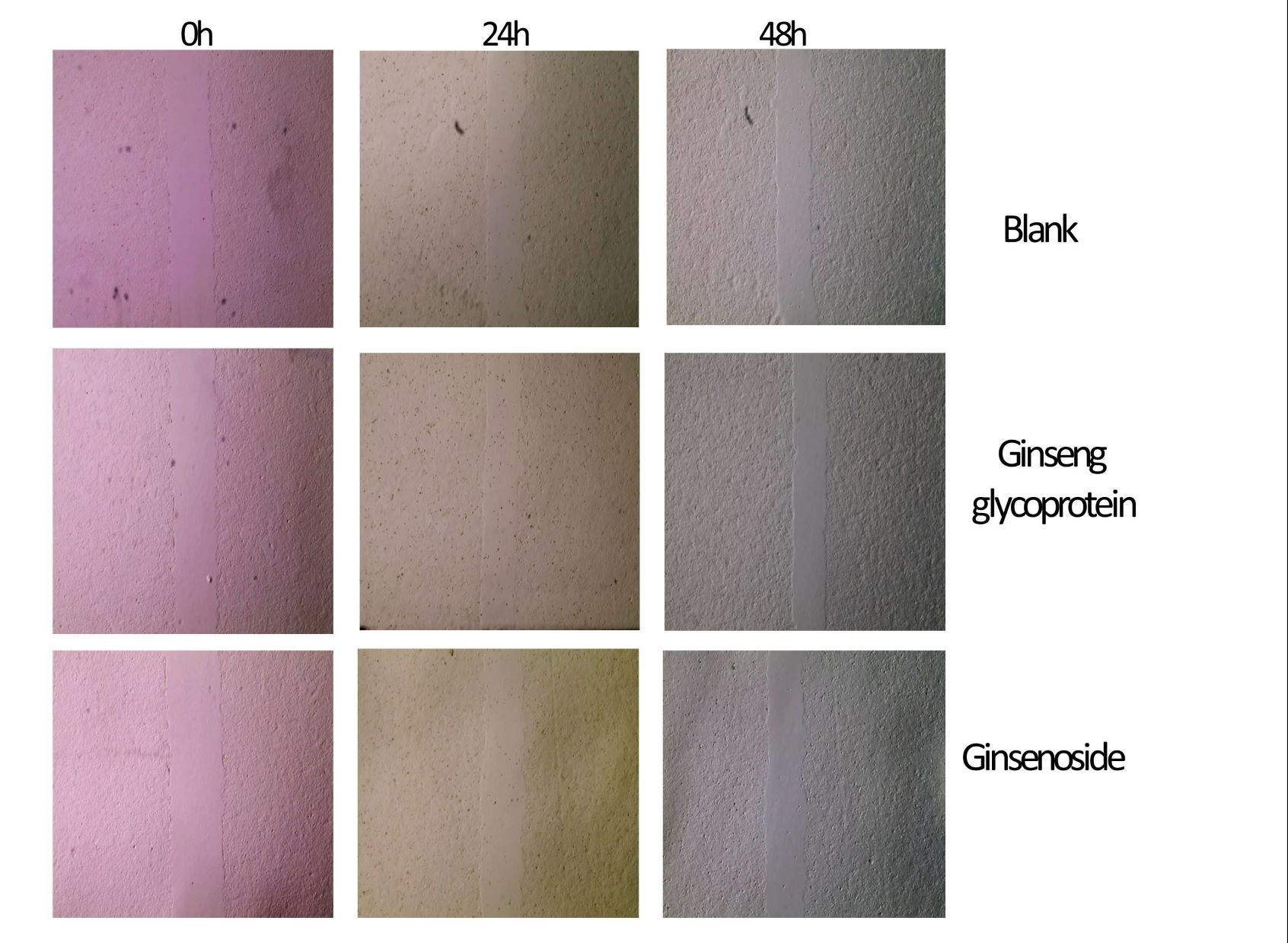

Supplement: Supplementary file 1 [file DataSheet1.ZIP › Original Date/Figure4/Figure4A.jpg]

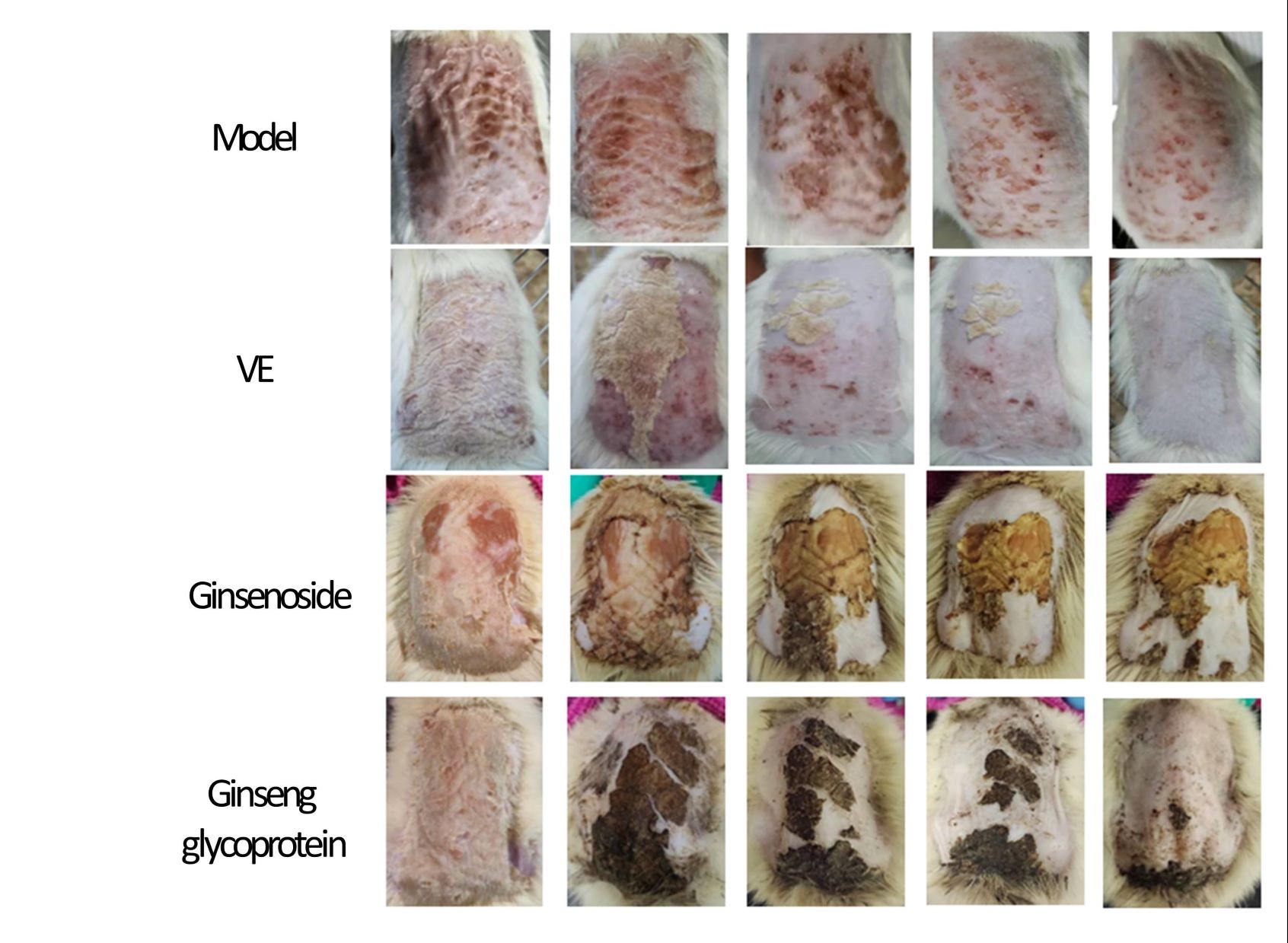

Supplement: Supplementary file 1 [file DataSheet1.ZIP › Original Date/Figure5/Figure5A.jpg]
